# Case Report: Fluzoparib combined Exemestane in gBRCA2-mutated HR+/HER2− advanced breast cancer

**DOI:** 10.3389/fphar.2025.1673418

**Published:** 2025-10-09

**Authors:** Dunya Yang, Xiaoyu Zhang, Xinyu Hu, Shaoqin Lin, Naying Yu, Xianglan Lin, Qunxiang Chen, Xi Chen

**Affiliations:** ^1^ Department of Oncology, 900th Hospital of PLA Joint Logistic Support Force, Fuzhou, Fujian, China; ^2^ Department of Oncology, Fuzong Clinical Medical College of Fujian Medical University, Fuzhou, Fujian, China

**Keywords:** fluzoparib, BRCA, mutation, breast cancer, PARP inhibitor, case report

## Abstract

This case study details a 57-year-old woman with heavily pretreated, hormone receptor-positive (HR+), HER2-negative advanced breast cancer harboring a pathogenic germline BRCA2 mutation. Following progression on multiple prior therapies including endocrine therapy combined with a CDK4/6 inhibitor, chemotherapy, and an antibody-drug conjugate (resulting in liver metastases), blood-based next-generation sequencing (NGS) identified the gBRCA2 variant alongside persistent high ER expression. Guided by these molecular findings, treatment was initiated with the domestically developed, highly selective PARP inhibitor (PARPi) Fluzoparib (300 mg orally twice daily) combined with the aromatase inhibitor Exemestane (25 mg orally daily). The regimen was well-tolerated, with manageable grade 1–2 adverse events (anemia, nausea, rash). Follow-up imaging demonstrated complete resolution of the hepatic metastases. The patient achieved a remarkably prolonged progression-free survival (PFS) of 37 months on this combination therapy, representing the longest period of disease control in her metastatic course. Although eventual progression occurred (new axillary lymph node metastasis and suspected hepatic recurrence), this case demonstrates the exceptional efficacy and durable disease control achievable with Fluzoparib plus Exemestane in a pretreated patient with gBRCA2-mutated HR+/HER2-advanced breast cancer, highlighting a promising therapeutic approach for this molecularly defined population.

## Introduction

Approximately 5%–10% of breast cancer patients harbor germline BRCA1/2 mutations (Saj et al., 2024). These patients face distinct therapeutic challenges. While gBRCA mutation has been historically associated with triple-negative breast cancer, however, a significant proportion (up to 60%) presents with HR+/HER2− disease (O'Shaughnessy et al., 2020). These tumors often exhibit aggressive behavior and may develop resistance to conventional therapies. PARP inhibitors (PARPis), which exploit synthetic lethality in homologous recombination repair (HRR)-deficient cells due to BRCA mutations, represent a major therapeutic advance (Albarrán et al., 2023). The FDA (Food and Drug Administration) has approved Olaparib and Talazoparib, and the NCCN (National Comprehensive Cancer Network) guidelines (Category 1) recommend them for treating HER2− advanced breast cancer with gBRCA1/2 mutations (Kang et al., 2025; Hennes et al., 2020).

Fluzoparib is a highly selective PARP1/2 inhibitor developed in China (Wang et al., 2019). Although preclinical data supports its potent activity (Wang et al., 2019), real-world clinical evidence in advanced breast cancer, particularly combined with endocrine therapy in the HR+/HER2− gBRCA-mutated subtype, remains scarce. This case details the molecularly guided use of Fluzoparib plus Exemestane in a patient with HR+/HER2− advanced breast cancer and a gBRCA2 mutation, achieving remarkable long-term disease control. This case exemplifies how molecular diagnostics directly inform treatment sequencing and align with NCCN principles. It also highlights a promising strategy that may be relevant for future guideline updates.

## Case presentation

In 2015, a 57-year-old woman presented with a left breast mass and biopsy confirmed invasive ductal carcinoma. Immunohistochemistry (IHC) analysis revealed ER positivity at 95%, PR positivity at 30%, HER2 score of 1+ with FISH (fluorescence *in situ* hybridizatio) negative, and Ki-67 proliferation index of 35%. The tumor was staged as pT1cN2a.m.0 (IIIA), classified as Luminal B (HER2−). She underwent left modified radical mastectomy (2015), followed by adjuvant chemotherapy (Epirubicin + Cyclophosphamide - Taxane, EC-T), radiotherapy, and endocrine therapy (Leuprorelin plus Toremifene). The patient underwent bilateral salpingo-oophorectomy as castration in 2017; Toremifene continued until Jul. 2019 with a disease-free survival (DFS) of 51 months.

In Jul. 2019, multiple lung nodules were detected. VATS (Video-Assisted Thoracic Surgery Lobectomy resection) confirmed metastatic breast cancer (IHC: CK7+, Mammaglobin+, ER/PR retained). First-line therapy for advanced breast cancer was Fulvestrant plus Palbociclib (Sep. 2019-December 2020; PFS: 15 months). Upon bone progression (T11 vertebra, Dec. 2020), second-line therapy with Nab-paclitaxel plus Capecitabine (Dec. 2020-April 2021; 7 cycles) followed by Capecitabine maintenance (Apr. 2021-February 2022; PFS: 14 months total) was administered.

**FIGURE 1:**
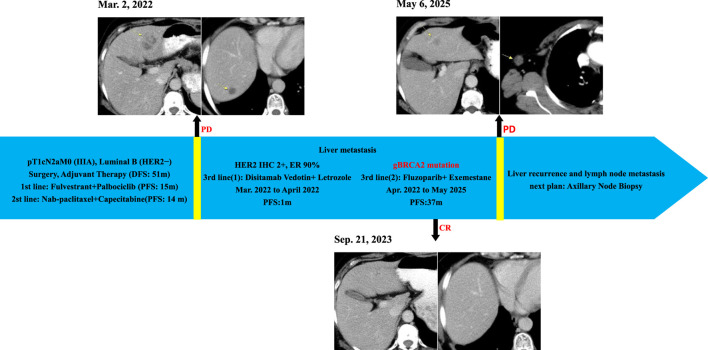
Clinical Course and Imaging Surveillance Timeline. 1) Contrast-enhanced CT (Mar. 2, 2022) showed multiple enhancing lesions consistent with hepatic metastases; 2) Contrast-enhanced CT (Sep. 21, 2023) demonstrated no evidence of hepatic metastasis; 3)Contrast-enhanced CT (6 May 2025) showed suspected recurrent metastatic lesion in the left hepatic lobe and metastatically enlarged right axillary lymph node.

**FIGURE 2:**
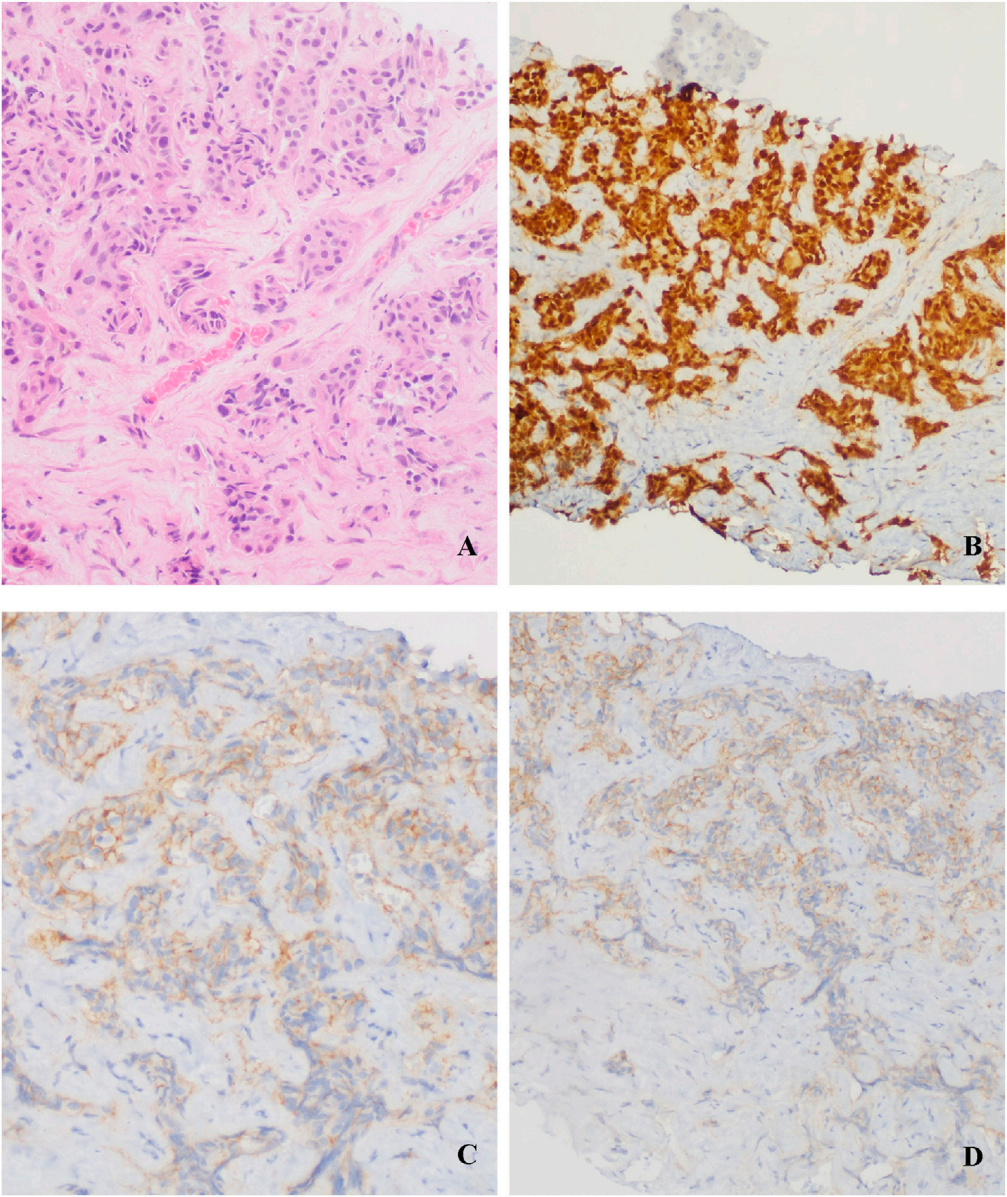
Liver metastasis biopsy: **(A)** Invasive carcinoma (HE staining); **(B)** ER 90%; **(C) (D)** HER2 IHC 2+.

In Feb. 2022, abdominal CT suggested multiple liver metastases (Figure 1). Subsequently, the patient presented to our hospital. Ultrasound-guided biopsy confirmed metastatic breast cancer (IHC: ER 90%, PR 1%, HER2 IHC 2+) (Figure 2). FISH for HER2 was negative. Due to HER2 IHC 2+, Disitamab Vedotin was initiated as third-line therapy (March 2022-April 2022). Concurrently, Letrozole was added based on high ER expression. After two cycles, CT showed minimal response.

In Apr. 2022, blood-based NGS detected a pathogenic gBRCA2 exon 11 variant (c.6462_6463del; p.Q2157Ifs*18; VAF 49.7%), confirming HRR deficiency. Based on this molecular evidence combined with persistent high ER expression—and considering the patient’s preference for oral therapy and economic factors favoring domestically developed agents—treatment was transitioned to oral Fluzoparib (300 mg twice daily) plus Exemestane (25 mg daily) in Apr. 2022.

## Results of molecularly guided therapy

Follow-up imaging (CT) demonstrated complete resolution of the previously identified liver metastases (Figure 1). The patient achieved a PFS of 37 months on Fluzoparib plus Exemestane. This duration represents the longest period of disease control in her metastatic course. Treatment was well-tolerated. Adverse events included: anemia (CTCAE Grade 2, managed with intermittent Erythropoietin), nausea (CTCAE Grade 1, managed with Ondansetron prn), and rash (CTCAE Grade 1, managed with topical steroids). No dose reductions or treatment interruptions were required.

Disease progression occurred in May 2025, evidenced by a new right axillary lymph node metastas and possible recurrence in the left liver lobe on CT (Figure 1), meeting RECIST 1.1 criteria. Planned evaluations include liver MRI, biopsy of the axillary node for IHC (ER, PR, CerbB2, PD-L1, MMR, TROP2) and genetic analysis (BRCA1/2 reversion, HER2, PIK3CA, ESR1) to guide next-line precision therapy.

## Discussion

This case powerfully illustrates the impact of molecular diagnostics on clinical outcomes in advanced breast cancer. The identification of a gBRCA2 mutation through timely blood-based NGS upon liver metastasis progression was the pivotal factor enabling a highly effective, targeted therapeutic strategy. The selection of Fluzoparib plus Exemestane, driven by the molecular finding of HRR deficiency (gBRCA2 mutation) and sustained HR positivity, yielded an exceptional PFS of 37 months in the fourth-line setting. This result significantly exceeded the median PFS reported for single-agent Olaparib (7.0 months) and Talazoparib (8.6 months) in registration trials for gBRCAm advanced breast cancer (Robson et al., 2017; Litton et al., 2018), and greatly surpassed the benefit seen with prior treatment lines in this patient.

The profound and durable response observed underscores the central role of synthetic lethality achieved by PARPis in BRCA-deficient cells (Faraoni and Graziani, 2018). Fluzoparib’s high selectivity for PARP1/2 potently inhibits base excision repair, leading to accumulation of DNA double-strand breaks that cannot be repaired in HRR-deficient tumor cells (Kutuzov et al., 2021). The synergy with endocrine therapy (Exemestane) is mechanistically plausible because PARPi-induced DNA damage may impair the ER pathway, and ER blockade can downregulate critical DNA repair genes (e.g., BRCA1, RAD51), together creating a “dual hit” that exacerbates genomic instability and enhances tumor cell death in HR + disease (Sottnik et al., 2025; Wu et al., 2020). This combination strategy warrants dedicated clinical investigation, particularly in the HR+/HER2−/gBRCAm subtype which constitutes a substantial fraction of gBRCAm advanced breast cancer.

This case carries significant implications for the guideline-aligned practice:

1. Timeliness of Molecular Testing: The gBRCA2 mutation was detected only after three lines of metastatic therapy. The NCCN guidelines strongly recommend gBRCA1/2 testing for all patients with HER2− advanced breast cancer at diagnosis (Brugioni et al., 2023). Earlier detection could have allowed PARPi initiation at an earlier line, potentially maximizing the benefit, as suggested by exploratory analyses of the OlympiAD trial (Im et al., 2020). This case reinforces the critical recommendation for prompt gBRCA testing at advanced breast cancer diagnosis.
2. Therapeutic Options for gBRCAm HR+/HER2− advanced breast cancer: While PARPi monotherapy is standard, this case provides compelling real-world evidence supporting the efficacy and tolerability of Fluzoparib within this molecularly defined population. Furthermore, it highlights the potential of combining PARPis with endocrine therapy in HR + disease. Additionally, Fluzoparib offers a valuable option, particularly in regions where access to or cost of other PARPis is a barrier. This data supports the consideration of Fluzoparib within the treatment framework and encourages exploration of PARPi/endocrine therapy combinations in clinical trials and guidelines.
3. Overcoming Resistance & Future Directions: The eventual progression after 37 months suggests acquired resistance mechanisms, possibly including BRCA2 reversion mutations, restoration of HRR, or activation of bypass pathways (e.g., PI3K/AKT/mTOR) (Seed et al., 2024). Planned re-biopsy and genomic profiling align with the principles recommending repeat molecular testing at progression to guide therapy. This case underscores the need for research into optimal sequencing and combination strategies post-PARPi progression.

## Conclusion

This molecularly guided case demonstrates exceptional efficacy (PFS: 37 months) and tolerability of the combination of Fluzoparib and Exemestane in a patient with heavily pretreated HR+/HER2− advanced breast cancer harboring a gBRCA2 mutation. The pivotal role of blood-based NGS in identifying the targetable gBRCA2 alteration directly enabled this successful therapeutic strategy. The profound clinical benefit achieved significantly surpasses outcomes typically seen with approved PARPis in similar settings and highlights the synergistic potential of combining PARPis with endocrine blockade in HR + gBRCAm disease.

This experience strongly reinforces the guideline recommendation for prompt gBRCA1/2 testing in all patients with HER2− advanced breast cancer at diagnosis, because earlier identification could optimize treatment sequencing. It provides robust real-world evidence supporting the clinical utility of Fluzoparib as an effective PARPi option for gBRCA-mutated advanced breast cancer. The remarkable PFS observed warrants further clinical investigation of Fluzoparib, particularly in combination with endocrine therapy, for HR+/HER2− gBRCAm advanced breast cancer, potentially informing future refinements to treatment algorithms. This case exemplifies the transformative impact of molecular insights on precision oncology.

## Data Availability

The datasets presented in this study can be found in online repositories. The names of the repository/repositories and accession number(s) can be found in the article/supplementary material.
